# Designing Surface and Interface Structures of Copper-Based Catalysts for Enhanced Electrochemical Reduction of CO_2_ to Alcohols

**DOI:** 10.3390/ma17030600

**Published:** 2024-01-26

**Authors:** Yanbo Hua, Chenyuan Zhu, Liming Zhang, Fan Dong

**Affiliations:** 1 Yangtze Delta Region Institute (Huzhou), University of Electronic Science and Technology of China, Huzhou 313001, China; 2 Shanghai Key Laboratory of Molecular Catalysis and Innovative Materials, Department of Chemistry, iChEM (Collaborative Innovation Center of Chemistry for Energy Materials), Fudan University Shanghai, Shanghai 200438, China; 3 Research Center for Environmental and Energy Catalysis, Institute of Fundamental and Frontier Sciences, University of Electronic Science and Technology of China, Chengdu 611731, China

**Keywords:** electrochemical CO_2_ reduction, copper-based catalysts, alcohols, mechanism understanding

## Abstract

Electrochemical CO_2_ reduction (ECR) has emerged as a promising solution to address both the greenhouse effect caused by CO_2_ emissions and the energy shortage resulting from the depletion of nonrenewable fossil fuels. The production of multicarbon (C_2+_) products via ECR, especially high-energy-density alcohols, is highly desirable for industrial applications. Copper (Cu) is the only metal that produces alcohols with appreciable efficiency and kinetic viability in aqueous solutions. However, poor product selectivity is the main technical problem for applying the ECR technology in alcohol production. Extensive research has resulted in the rational design of electrocatalyst architectures using various strategies. This design significantly affects the adsorption energetics of intermediates and the reaction pathways for alcohol production. In this review, we focus on the design of effective catalysts for ECR to alcohols, discussing fundamental principles, innovative strategies, and mechanism understanding. Furthermore, the challenges and prospects in utilizing Cu-based materials for alcohol production via ECR are discussed.

## 1. Introduction

The carbon-neutral production of fuels and chemical feedstocks is one of the grand challenges for our society to solve [1]. The conversion of CO_2_ into value-added fuels is particularly beneficial for establishing a carbon-neutral system, resulting in widespread interest. Various methods for converting CO_2_ into carbon-containing fuels exist, including thermochemical, photochemical, biochemical, and electrochemical catalytic conversion routes [2,3,4]. Among these routes, electrochemical CO_2_ reduction (ECR) has gained significant attention in recent years due to its advantages in terms of operating under ambient temperature and pressure conditions, as well as its simplicity in operation. Furthermore, the utilization of electricity from renewable energy sources, such as solar, wind, and tidal power, provides an effective approach for energy storage and conversion to address the challenges posed by geographical and intermittent renewable energy availability. However, CO_2_ poses a significant challenge in its conversion to other compounds due to its exceptional stability and the high energy required to break the C-O bond (about 750 kJ mol^−1^) [5]. The inherent stability and chemical inertness of linear CO_2_ contribute to the uphill energy process and high activation barrier, leading to large overpotentials and limiting the efficiency of CO_2_ conversion. Additionally, the hydrogen evolution reaction (HER) competes with ECR and further hampers selectivity towards carbonaceous products [6].

It was reported that various target products can be obtained from CO_2_ by means of ECR, for instance, CO, HCOOH, CH_4_, CH_3_OH, C_2_H_4_, and so on. Among the many products that can be produced by means of ECR, alcohols (CH_3_OH, C_2_H_5_OH, C_3_H_7_OH, etc.), with a high market price and a large market size, are attractive targets [7]. Alcohols hold a prominent position in modern society as vital organic commodity chemicals, finding extensive applications as fuel components, chemical synthesis precursors, and essential compounds in the medical and food industries. In conventional industries, the production of alcohols needs to use agricultural feedstocks and consume a large amount of thermal energy. In contrast, selective ECR to alcohols with renewable electricity is a green and sustainable route, which is highly desirable.

Due to its unique characteristics and properties, copper (Cu) stands out as the sole single-metal catalyst capable of generating high-energy-density hydrocarbons and alcohols with reasonable efficiencies [8]. Numerous Cu-based catalysts have been reported to facilitate the production of specific alcohols through ECR. However, the ECR process for alcohol production still faces several challenges, including high overpotential, low Faradaic efficiency (FE), and low yield rates. Moreover, the inert nature of the CO_2_ molecule and the involvement of multiple electron and proton transfers render the overall reaction kinetically sluggish, necessitating large overpotentials for both the anodic oxygen evolution reaction (OER) and the cathodic CO_2_ reduction [8]. Hence, designing electrochemical catalysts for efficient ECR to alcohols with high selectivity and low overpotential is crucial.

While recent progress in ECR for alcohol production, particularly that of ethanol, has been extensively reviewed, significant achievements have also been made in developing catalytic materials for ECR towards multicarbon alcohols [9,10]. Thus, a comprehensive review of the state of the art in advanced catalyst design for alcohol production from ECR is warranted. This review article aims to guide further research by discussing the fundamental principles of catalyst design and the mechanisms involved in alcohol production. Firstly, we delve into the mechanism leading to alcohols as the fundamental principle for designing catalysis materials. Subsequently, we extensively review innovative strategies based on newly developed electrocatalysts, followed by a discussion on advanced spectroelectrochemical analysis. Finally, we address the remaining challenges and provide perspectives for ECR to alcohols. We believe that this critical minireview will provide essential background information for further advancements in the applications of Cu-based materials in ECR for alcohol production.

## 2. Fundamentals for CO_2_ Reduction to Alcohols

Understanding the specific reaction pathways of ECR to alcohols is of utmost importance in guiding the design and synthesis of highly efficient catalysts. However, the process of alcohol generation via ECR consists of multiple charge-transfer steps, requiring 6 to 18 electrons and protons in total [7] (Table 1). Moreover, the overlapping energy levels between ECR and the hydrogen evolution reaction (HER) make mechanistic studies of ECR more difficult, leading to some unanswered mechanistic questions for the field. Therefore, various techniques like spectroscopy and electrochemical analysis, along with theoretical calculations, have been used to investigate the reaction pathways leading to alcohol formation in ECR [11,12]. These studies help us to understand the role of catalyst materials, surface structures, and reaction conditions in influencing alcohol production.

**Figure 1:**
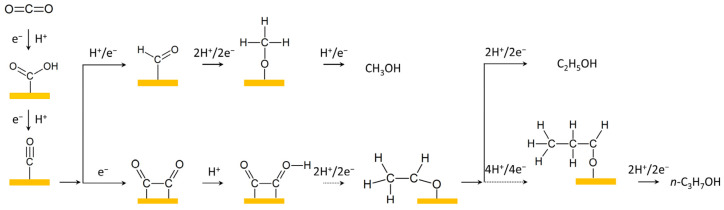
The reaction pathways of electrocatalytic CO_2_ reduction to various alcohols.

Figure 1 depicts a simplified representation of a widely accepted pathway for alcohol formation in ECR. The actual mechanisms may be more complex, involving additional reactions and intermediate species [14,15]. Further research is needed to fully understand these pathways and optimize catalyst design for better alcohol production. Currently, the ECR process can be dissected into three pivotal stages: the formation of a CO intermediate, C-C coupling, and the hydrodeoxygenation of C_2_ intermediates [16,17]. Initially, the CO_2_ molecule is adsorbed onto the catalyst surface and undergoes activation, resulting in the formation of adsorbed carbon dioxide (*CO_2_). Subsequently, a reduction reaction takes place, giving rise to the generation of adsorbed CO (*CO). It has been postulated that *COOH serves as the initial intermediate for CO formation, while *OCHO is deemed as the probable intermediate for formic acid production [18,19]. In-depth investigations employing in situ surface-enhanced infrared absorption and Raman spectroscopic techniques have shed light on the essential role played by the *CO species in facilitating the production of >2e^−^ products during ECR [20,21]. Achieving an optimal binding strength of the *CO species is of paramount importance in promoting alcohol formation and facilitating the C-C coupling process, particularly in the context of ethanol and n-propanol production.

The production of the C_1_ alcohol, methanol (MeOH), involves the protonation of *CO, leading to the formation of the absorbed formyl (*COH) intermediate, which represents the rate-determining step (RDS). Subsequently, *COH undergoes a cascade of proton–electron coupled transfer (PECT) steps, ultimately yielding the *OCH_3_ species. The selectivity between MeOH and methane hinges on the subsequent hydrogenation of *OCH_3_. For the formation of C_2+_ alcohols, the C-C coupling process assumes a pivotal role. The generation of ethanol entails a rate-determining step (RDS) identified as *CO-CO dimerization, followed by protonation and dehydration steps, ultimately leading to the formation of the intermediate *CH_2_-CHO. The subsequent reduction pathway of *CH_2_-CHO can bifurcate, resulting in the production of either ethylene or C_2+_ alcohols. Consequently, *CH_2_-CHO assumes the role of the selectivity-determining intermediate (SDI) governing the production of C_2+_ alcohols. The production of C_3_ alcohols (PrOH) through ECR remains an ongoing challenge, with limited success reported in obtaining C_3_ alcohols. Proposed mechanisms involve the intermolecular C-C coupling of adsorbed C_2_ and C_1_ intermediates, followed by intricate proton–electron transfers, ultimately leading to the formation of propionaldehyde. Accordingly, propionaldehyde can be further hydrogenated to produce PrOH [22,23,24]. In addition to the above pathway from *CO-CO dimerization, it was also found that ethanol can be selectively enhanced via the *CHx-*CO coupling pathway on a Cu surface in a CO-enriched environment [25,26].

## 3. Strategies to Improve Alcohol Production

### 3.1. Crystal Facet Regulation

**Figure 2:**
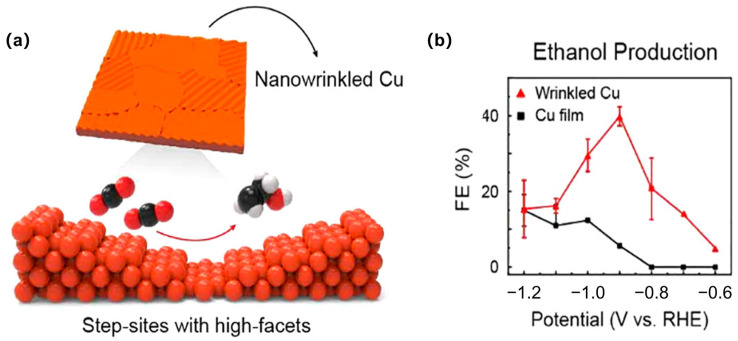
(**a**) Schematic of the synthesis of a highly dense Cu step-site catalyst with a high-facet atomic arrangement. (**b**) Faradaic efficiency results in 0.1 M KCl electrolyte by varying the potential with winkled Cu and Cu film catalysts. Reproduced with permission from Ref. [36], Copyright (2021) ACS.

As a typical model system, single-crystal materials have been paid great attention for their structure–performance relationship in ECR and many other catalysis systems [7,27]. Due to the distinct arrangement of surface atoms and the resulting interaction with reaction molecules, different crystal facets of the catalysts tend to present varied performance toward ECR [28]. The first ECR on single-crystal Cu was performed by Frese, who found increasing CH_4_ generation on Cu(100), Cu(110), and Cu(111) surfaces [29]. In 2002, Hori et al. systematically studied the important impact of Cu facets toward specific ECR products, including CH_4_, C_2_H_4_, CH_3_COOH, CH_3_CHO, and C_2_H_5_OH [30]. So far, a number of studies on copper single crystals have deepened our understanding of the structure–activity relationship of specific crystal facets for ECR. For example, Cu(100) was found to be easier for C-C coupling by combining electrochemical tests and DFT calculations [31]. In situ Raman was performed recently, confirming that higher surface coverage of adsorbed *CO on the Cu(110) surface promotes the formation of the *OCCO and *CH_2_CHO intermediates to generate C_2_ products; comparatively, the Cu(111) surface possessed low *CO coverage to produce CH_4_ [32]. In recent research, a product-specific active site for ECR was concluded by means of detailed analysis on nine single-crystal copper surfaces. The functions of lattice facet, coordination number, and step-terrace angle were taken into consideration for specific ECR performance, and Cu(110), which possesses a coordination number of seven and a larger step-terrace angle, was found to be able to promote ethanol production [33]. Additionally, some high-index copper facets have been found to prefer C_2+_ production in ECR [34,35]. For example, a wrinkled Cu catalyst with high-density (200) and (310) facets was fabricated by means of a chemical vapor deposition (CVD) graphene growth process (Figure 2). High ethanol selectivity of 40% was achieved at −0.9 V vs. RHE during ECR, and the (310) facet was calculated to possess a low C-C coupling barrier and preferred ethanol pathway [36]. By covering Cu overlayers on THH Pd NCs with high-index facets, ∼20% FE of ECR to ethanol at −0.46 V vs. RHE was obtained [37].

### 3.2. Oxide-Derived Cu

Generally, the surface of copper can be oxidized easily without protection [38]. The extraordinary performance of oxide-derived copper (OD Cu) in catalyzing CO_2_ into deeply reduced products has already been found. In 1990, Frese et al. noticed that the production of MeOH can be promoted on Cu_2_O, which they attributed to the role of Cu(I) or monolayer oxygen [39], leading the research on OD Cu toward deep ECR/CORR [40,41,42].

**Figure 3:**
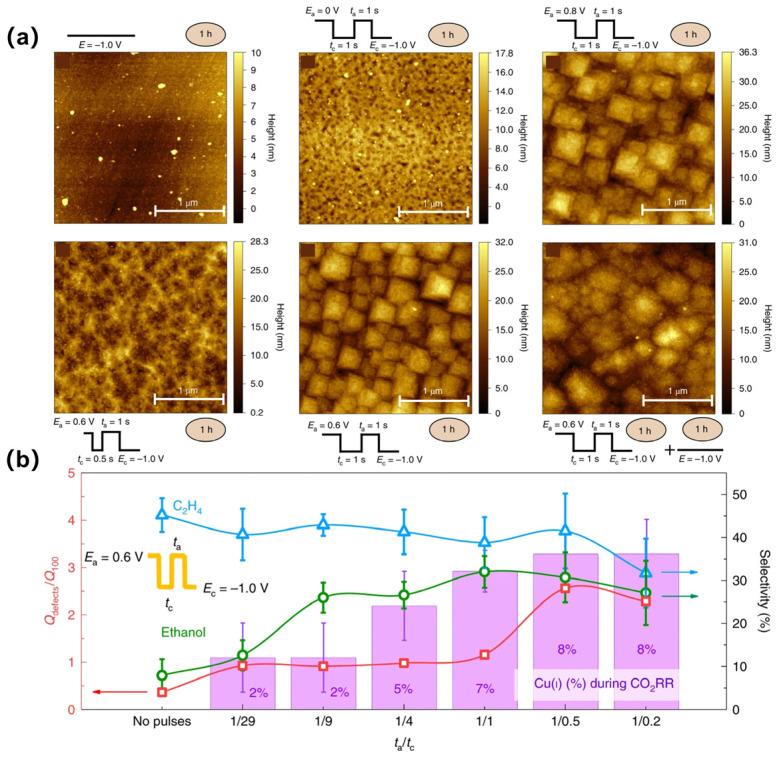
(**a**) Atomic force microscopy images of a Cu(100) electrode after different surface treatments and reaction settings. (**b**) Q_defects_/Q_100_ and product selectivity as a function of t_a_/t_c_ applied. Reproduced with permission from Ref. (The red arrows correspond to Q_defects_/Q_100,_ The blue arrows correspond to the selectivity of CH_4_, The green arrows correspond to the selectivity of ethano) [51], Copyright (2020) Springer Nature.

The function of the oxidative copper or oxygen in OD Cu for catalyzing deep ECR was further studied [43,44,45]. Strategies have also been proposed to maintain the positive state of copper during ECR, such as by adding self-sacrificing supports or electron receivers [46,47]. Doping boron with Cu is an efficient approach to tune and increase the stability of Cu^δ+^ under ECR. By incorporating B atoms, boron-doped copper exhibits stable electron localization, leading to the production of highly selective ethylene and ethanol products [48,49,50]. Using a pulsed electrolysis technique that intermittently applies a suitable positive potential during negative potentiostatic electrolysis, significantly enhanced ethanol production on copper was discovered. It was believed that the coexistence of the continuously in situ regenerated Cu(I) with Cu(0) species helped improve the CO_2_-to-ethanol performance [51] (Figure 3a,b). Chen et al. also suggested that Cu and Cu(I) can offer an asymmetrical OCCO adsorbing site, ensuing the stabilization of the carbonyl group by the OH groups at the boundary of Cu-Cu(I) motifs, promoting the formation of asymmetric alcohols [52]. The positive polarization of the electrode also lowers the coverage of the surface hydrogen, thus suppressing HER and improving ethanol formation due to the higher OH concentration. Some calculations also concluded the function of the special interface of Cu(I) and Cu(0) to improve the kinetics and thermodynamics of both CO_2_ activation and CO dimerization [53]. First, Cu^+^ sites can bind an H_2_O molecule neighboring to the Cu^0^ region, which can form strong hydrogen bonds with the absorbed CO_2_ on the Cu^0^, stabilizing both the transition state and the final state. Second, when there are nearby Cu^+^ and Cu^0^ that the respective C atoms of two CO can bond with, the C atom of CO@Cu^+^ is positively charged, while the C atom of CO@Cu^0^ is negatively charged due to back donation. The attractive electrostatics between the two C atoms facilitate C-C coupling.

On the other side, some researchers believed that it is hard to maintain the positive state of Cu in OD Cu under the negative potential of ECR, so the genuine active site for deep ECR cannot be the positively charged Cu. As some studies showed, the oxygen in copper oxide was completely removed during ECR, and the real active sites for C_2+_ production were the subsequently generated low-coordinated copper sites and abundant grain boundaries that improve C-C coupling [54,55,56]. This suggests that we should follow the state of Cu during ECR in detail using in situ/operando techniques to draw a solid conclusion.

### 3.3. Alloying

The introduction of heteroatoms into copper, either to adjust the electronic structure, to promote specific intermediate adsorption, or to protect active sites and generate synergies, can sometimes obtain superior ECR performance to that of pure copper metal [57,58,59]. Alloys can be thought of a special kind of doping material that possesses relatively uniform crystal structure. Outstanding performances have also been gained on copper-based alloys. For example, a series of Pd_x_Cu_y_ bimetallic aerogels with varied compositions were fabricated. The selectivity of MeOH generation during ECR was found to correlate with the atom ratio of Pd and Cu. An extremely high FE of 80.0% for MeOH with a current density of 31.8 mA cm^−2^ was obtained with Pd_83_Cu_17_. This outstanding performance was credited to the high Pd^0^/Pd^II^ and Cu^I^+Cu^0^/Cu^II^ ratios and sufficient Pd/Cu grain boundaries, but the underlying mechanism needs to be further explored [60]. Au and Ag are more frequently chosen as alloy metals with Cu. Au-Cu alloy nanoparticle-embedded Cu submicrocone arrays were designed for ECR, and 29 ± 4% selectivity for ethanol was gained [57]. It was stressed that the Au can regulate the binding energies of key intermediates (including CH_2_CHO*, CH_3_CHO*, and CH_3_CH_2_O*), so the activity and selectivity of EtOH/C_2_H_4_ can be adjusted through controlling the content of Au. Recently, a CuAg alloy catalyst was obtained by means of co-electrodeposition in a supersaturation environment. Under supersaturated conditions in highly carbonated electrolytes, the alloy presented a high selectivity of ECR to 2-propanol, with an FE of 56.7% and a specific current density of 59.3 mA cm^−2^. Operando FTIR suggested the critical role of *CO and *OCH_2_CH_3_ for C_1_-C_2_ coupling, as the potential decreased from −0.2 to −0.73 V vs. RHE, and both their bands were progressively intensified. Further calculations showed that the surface binding of intermediates in the middle position of the alkyl chain was weakened, while the C-O bonds were strengthened due to the dispersed Ag atoms in Cu, facilitating the formation of 2-propanol over 1-propanol [61].

### 3.4. Tandem Catalysis

**Figure 4:**
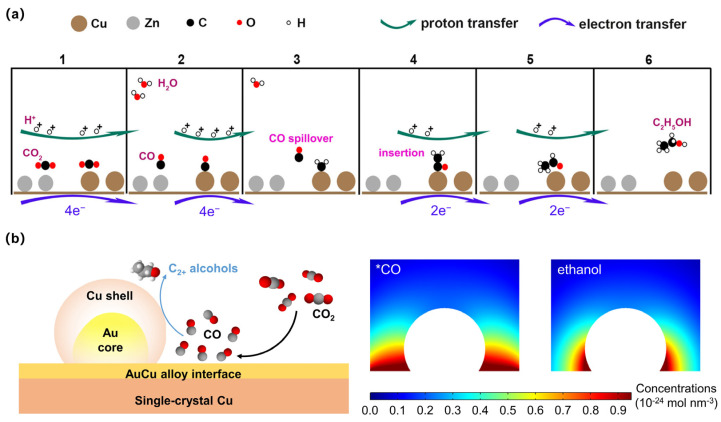
(**a**) Tandem mechanism for the electroreduction of CO_2_ to ethanol on Cu_x_Zn catalysts: stages 1 → 2, four protons and four electrons reduce two CO_2_ molecules to CO on Cu and Zn, respectively; stages 2 → 3, four protons and four electrons reduce CO molecule to *CH_2_ on Cu, while CO produced by Zn desorbs and migrates near the *CH_2_; stages 3 → 4, CO inserts into the bond between Cu and *CH_2_ to form *COCH_2_; stages 4 → 5, two protons and two electrons reduce *COCH_2_ to CH_3_CHO (acetaldehyde); stages 5 → 6, two protons and two electrons reduce CH_3_CHO to CH_3_CH_2_OH (ethanol). Reproduced with permission from Ref. [63], Copyright (2016) ACS. (**b**) A schematic diagram of the “tandem” electrocatalysis pathway on a reconstructed Au-Cu electrode (**left**), and an overview of the concentration and flux distribution of *CO and ethanol on a reconstructed Au-Cu heterostructure (**right**). Reproduced with permission from Ref. [67], Copyright (2022) Elsevier Inc.

In a tandem catalysis system, there may be two or more kinds of components working in turn in different steps toward deep ECR for C_2+_ products. Due to the better performance of CORR in generating C_2+_ products as compared to ECR on copper, the introduction of an assisting metal to produce CO for copper is an promising strategy for deep ECR [62]. For example, Zn was introduced to produce CO, which could then migrate to copper to form *CH_x_. The *COCH_2_ formed after further CO insertion served as an intermediate to obtain alcohols [63] (Figure 4a). In another work, gold nanoparticles were deposited on a polycrystalline copper foil surface, and greatly enhanced C_2+_ alcohol production was obtained due to the high CO concentration generated by gold and the further reduction on copper in a locally alkaline environment [64].

Besides Zn and Au, Ag has also been chosen as the assisting component in tandem systems. A specially designed Cu@Ag core–shell NP structure was reported for tandem catalysis. The production of CO and C-C coupling was realized on the Ag shell and Cu core, respectively, offering inspiration for catalyst structure design for tandem systems [65]. In another work, the importance of efficient CO intermediate management for tandem catalysis was stressed. A segmented gas-diffusion electrode (s-GDE) was designed to integrate an inlet CO-selective catalyst layer (CL) segment and a subsequent C_2+_-selective segment. By adjusting the relative lengths and loadings of the two parts (e.g., Ag and Cu), the residence time of CO in the Cu CL segment can be maximized. Compared to a non-segmented Cu/Ag GDE, a 300% increase in CO utilization was achieved, and a 250% increase in jC_2+_ relative to pure Cu was gained [66].

More detailed reaction observations and calculations, together with device engineering, have been carried out to understand the structure–activity relationship and obtain better performance for ECR tandem catalysis. Taking an epitaxial Au/Cu heterostructure as a model system, Zhu et al. found that the restructured Au-Cu alloy supported Au@Cu core–shell nanoclusters during ECR under atomic-resolution TEM, which was driven by Au interdiffusion and Cu redeposition (Figure 4b). The in situ formed Au-Cu alloy was thought to provide active sites for the stable generation of CO, which was further reduced to C_2+_ alcohols on the Cu shell, as proved by finite-element simulation and DFT calculations. The catalyst had ∼150 mV more positive onset potential toward C_2+_ alcohols and presented a 400-fold improvement in the generation of alcohols over hydrocarbons compared to monometallic Cu [67]. In another work on copper–gold heterojunctions, a 60% FE of ethanol at a current exceeding 500 mA cm^−2^ was achieved, and the critical function of the intermediate was stressed. In situ ATR-IR measurements and simulations suggested that reduction of CO_2_ at the copper/gold heterojunction is dominated by the production of the OCCOH* intermediate, the asymmetrical hydrogenation of which leads to superior selectivity toward ethanol [68].

### 3.5. Single-Atom Catalysts

Generalized single-atom catalysts (SACs) include molecular catalysts, carbon- or metal-oxide-supported single-metal-site catalysts, and some dispersed metal alloys [69]. ECR has been broadly studied on these materials, and some of them have shown outstanding performance. For example, with carbon nanotubes as a conductive support, cobalt phthalocyanine (CoPc) presented great potential for methanol production [70,71]. As for Cu single atoms, Yang et al. fabricated Cu-decorated through-hole carbon nanofibers (CuSAs/TCNFs), which presented 44% methanol production during ECR [72]. DFT calculations showed that the Cu single atoms could bind more strongly with the *CO intermediate, which could be further reduced rather than being easily released as CO. The abundant exposed Cu single atoms also endowed the catalyst with a −93 mA cm^–2^ partial current density for C_1_ products and 50 h stability. Recently, a >60% methanol FE was achieved using monodispersed cobalt phthalocyanine (CoPc) on single-walled CNTs (CoPc/SWCNTs) for ECR. Raman spectroscopy combined with XPS and XANES illustrated that the strong molecule–support interaction induced the local geometry and electronic structure change of the CoPc anchored on SWCNTs. Further calculations suggested that the curved CoPc can bind more strongly with *CO, making the deeper reduction to methanol of the latter easier compared to that of *CO on CoPc with low deformation [71].

Generally, the production of C_2+_ products by means of ECR on copper needs two nearby copper sites to realize C-C coupling, which appears to be hard for many SACs. However, it has been discovered that SACs can also make sense. For example, using a Cu-N_4_ structured catalyst based on a N-doped carbon matrix obtained via a pyrolytic route, 55% FE of ethanol was achieved by means of ECR under −1.2 V vs. RHE in 0.1 M CsHCO_3_ solution [73]. Operando XAS observations showed that the in situ Cu-Cu bond formed under the optimal catalytic potential, which implied that the Cu single atoms can migrate to generate Cu clusters to serve as the real active sites for ethanol production. This work inspires the notion that SACs can serve as precursors for real active site generation. In another work, ECR was conducted on a carbon-supported single-Cu-atom catalyst synthesized through a Cu-Li method [74]. High selectivity of 91% toward ethanol generation was obtained, and via operando XAS characterization, the initial dispersed Cu atoms were found to reversibly form Cu_n_ clusters in the applied electrocatalytic environment, acting as the genuine catalytic sites. A number of studies on Cu-based MOFs and molecular catalysts (e.g., CuPc and CuPor) combining experimental and theoretical calculations also found that the isolated Cu centers tended to aggregate, creating Cu nanoparticles to actually catalyze ECR to generated deep reduced products [75,76,77,78,79]. On the other hand, some researchers believe that the single Cu atoms can remain stable during the ECR process. In a recent work, Xia et al. synthesized Cu SACs with a Cu content of up to 13.35 wt% by means of a silica-mediated hydrogen-bonded organic framework (HOF)-templated strategy [80]. Electrochemical testing of ECR in an H cell found that under −1.1 V vs. RHE in 0.5 M CsHCO_3_, the FE of ethanol reached 81.9% with a partial current density of 35.6 mA cm^−2^. Further DFT calculations evidenced that the adjacent Cu-N_3_ structures serve as active sites to promote C-C coupling. However, due to the lack of operando observations of the Cu states, the real behavior of the Cu atoms during catalysis remained unclear. Taking the above phenomenon into consideration, the real structure–activity relationship of Cu-based single-site catalysts needs to be carefully considered.

### 3.6. Interface Engineering

Attaching groups or molecules to the copper surface or modifying the copper surface with additives is sometimes an effective way to regulate the catalytic performance to build a specific microenvironment. For ECR, some of the benefits that surface ligands can bring to catalysts were discussed in [81,82]. Ligands on copper can effectively adjust the surface concentration of intermediates and their interaction with the catalysts. For example, when Cu nanoneedles were coated with hydrophobic PTFE, the supply of protons to the catalysts and, thus, HER was suppressed, with ethanol production elevated from 7.7% to 25.8% due to the concentrated CO_2_ [83]. In another work, by modifying a sputtered copper surface with alkanethiols of different alkyl chain lengths to continuously regulate the interfacial wettability, the mass transport of CO_2_ and H_2_O during ECR was regulated. The resulting changes in *CO and *H coverage were quantified by means of in situ ATR-SEIRAS spectra and the decay distances from CLSM, revealing that the increase in hydrophobicity led to increasing *CO coverage and decreasing *H coverage. The variation in the kinetic-controlled *CO and *H ratio affected ethylene and ethanol pathways such that at the optimal level, a highest selectivity for ethanol of 53.7% was gained [84].

In another work, an h-BN/Cu interface was constructed, the perimeter of which was concluded to provide specific chelating sites to stabilize the intermediates, activating the conversion of *CO to *CHO; >60% CH_4_ formation was achieved during ECR [85]. As a surface modification to improve catalytic stability, graphene oxide was coated onto 5-fold twinned copper nanowires for ECR. Intensified morphological stability and CH_4_ production selectivity were obtained due to the protection provided by the GO [86]. Follow-up work should be carried out to investigate more delicate regulation of the catalyst surface ligands in order to realize optimal interaction with the reaction species, controllable catalytic performance, and improved stability.

### 3.7. Non-Metal Sites

Catalysts without a metal component can lower manufacturing costs and improve catalytic stability, given that metal loss and deactivation appear frequently in many metal catalysis systems. Due to their high electrical conductivity and structural stability, carbon materials are usually chosen as a support to dope nonmetal heteroatoms (e.g., N and B) for ECR [87,88]. In some special designed systems, efficient alcohol production was achieved using doped carbon. For example, Wu et al. designed N-doped graphene quantum dots (NGQDs) to catalyze CO_2_, reaching a high FE of ECR of up to 90% and a selectivity for ethylene/ethanol of 45%. Ex situ X-ray photoelectron spectroscopy (XPS) revealed that the pyridinic N located at the edge site of graphene accelerated the CO_2_ adsorption [89]. Subsequently, mesoporous N-doped carbons were fabricated to catalyze CO_2_ into EtOH with an FE of up to 77% at −0.56 V vs. RHE, in which pyridinic N was concluded to favor *CO formation for further C-C coupling to form ethanol [90]. Calculations also confirmed the reduced free energy of ECR to ethanol on N-doped graphene [91]. Besides N, B was also selected to modify carbon for effective ECR. The FE of ECR to MeOH reached 24.3% on boron-doped diamond (BDD) in an NH_3_ solution [92]. Further, the co-doping of B/N on diamond even improved the selectivity of ECR to ethanol up to 93.2% at −1 V vs. RHE. DFT calculations proved the synergistic effect of B and N, wherein the former intensified the CO_2_ capture through bonding with one O atom of absorbed CO_2_, and the latter facilitated *H transfer for hydrogenation [93]. Enormous potential remains in this field for future exploration.

## 4. Advanced Spectroelectrochemical Analysis for Mechanism Understanding

Combined with theoretical calculations, many advanced characterization techniques have been playing an important role in ECR observation and mechanism understanding, especially in situ/operando analysis [11,94]. Chen et al. called for the development of various complementary in situ/operando techniques for dynamic interface detection, aiming to present a comprehensive picture of interfacial electrocatalysis [12]. Among these techniques, identifying reaction intermediates using spectroscopic techniques during electrocatalysis can help to deduce the reaction pathway and provide an understanding of the reaction mechanism. For example, through isotope-labelled co-reduction experiments where ^13^CH_3_I and ^12^CO were respectively co-fed as the methyl and carbonyl sources, the asymmetric C-C coupling pathway on a Cu surface was confirmed [95]. In situ infrared and Raman spectroscopy are powerful tools for reaction intermediate recognition. In recent research, the evolution of the adsorption strength of the intermediates, including *O_2_CO, *OCOOH, *COOH, and *CO, was observed on a Cu(100) surface using in situ Raman spectroscopy, combined with the formation of nanoclusters, which may influence the ECR reactivity [96]. In the following, research toward reaction pathway recognition is briefly summarized, classified by different alcohol products.

### 4.1. Methanol

**Figure 5:**
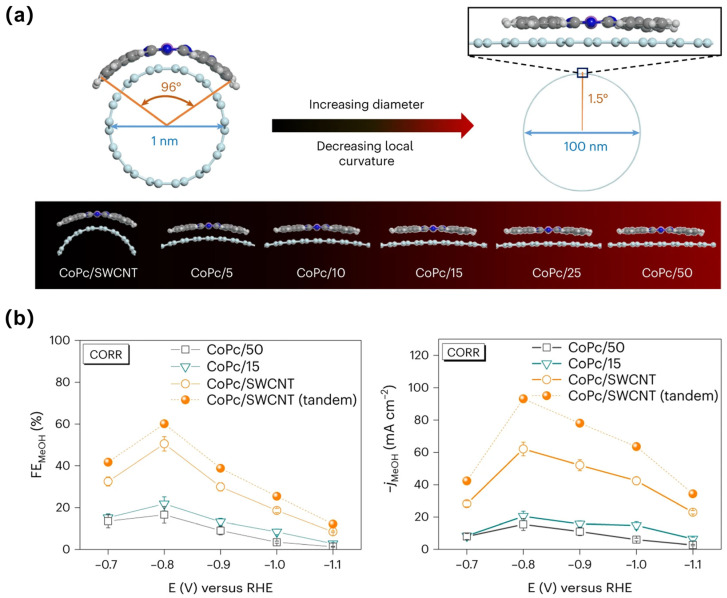
(**a**) Illustration of the structural distortion of CoPc on different-diameter CNTs, assuming that CoPc is fully elastic. (**b**) FE_MeOH_ and j_MeOH_ of CoPc/SWCNTs, CoPc/15, and CoPc/50 in a flow cell under CO atmosphere. Reproduced with permission from Ref. [71], Copyright (2023) Springer Nature.

Methanol is a valuable but relatively less desired product compared to ethanol in electrocatalytic ECR, and the research potential for efficient methanol production is huge. By immobilizing CoPc onto carbon nanotubes, 44% selectivity for methanol in six-electron ECR was obtained with a partial current density of 10.6 mA cm^−2^ at −0.94 V vs. RHE [70]. The pathway of methanol production on CoPc is thought to be a domino process in which CO_2_ first undergoes a two-electron reduction to CO, which is then reduced to MeOH through a four-electron–four-proton process. The superior catalytic activity of the catalyst is attributed to the individual dispersion state of CoPc molecules on highly conductive CNTs, helping in efficient electron transfer to the active site for multielectron reduction of CO_2_. Using monodispersed cobalt phthalocyanine (CoPc) on single-walled CNTs (CoPc/SWCNTs) for electrochemical CO conversion, a >60% methanol FE was achieved recently (Figure 5a,b) [71]. When CoPc is anchored on thin carbon nanotubes, the strong molecule–support interaction can induce a change in the local geometry and electronic structures of the catalyst. Raman spectroscopy showed the Co-N out-of-plane deformation and ring boating peak at 250–290 cm^−1^, while XPS showed the higher binding energies of Co 2p in CoPc/SWCNTs. The XANES results of Co presented the lower peak intensity of 1_s_→4P_z_, suggesting decreased symmetry of the CoPc molecule on SWCNTs. Further calculations suggested that the curved CoPc can bind more strongly with *CO, making the deeper reduction to methanol of the latter easier compared to that of *CO on CoPc with low deformation. In situ ATR-SEIRAS found a C-H stretching mode at ~3010 cm^−1^ for CoPc/SWCNTs, which may be from *OCH_2_ or *HOCH_2_. In comparison, no obvious signal was detected between 2600 and 3200 cm^−1^ for CoPc on 50 nm CNTs, which may be attributed to the poor *CO absorption hampering further reduction beyond *CO.

Ag, S dual-doped Cu_2_O/Cu was fabricated and presented 67.4% methanol production with a current density as high as 122.7  mA  cm^−2^ in an H cell [97]. S was thought to adjust the electronic structure and morphology of the catalyst to improve the methanol pathway, while Ag suppressed the HER. Their synergistic interaction was confirmed by comparing experiments and calculations, but direct characterization evidence is lacking. In another work, a Cu_2_NCN crystal with single-atom Cu sites and enhanced delocalization around Cu was successfully designed [98]. By applying the catalyst in ECR, 70% CO_2_-to-CH_3_OH selectivity and a current density of −92.3  mA  cm^−2^ were gained in an MEA-based electrolyzer. When applying the potential from −1.0 to −1.5 V vs. RHE, two Raman bands at 1080 and 1120 cm^−1^ were observed, respectively corresponding to *CHO and *OCH_3_, two key intermediates in the CH_3_OH pathway, suggesting that a CO_2_-to-CH_3_OH reaction occurred. Calculations showed that the softer Cu sites in Cu_2_NCN led to a weaker Cu-*OCH_3_ interaction than the O-CH_3_ interaction, leading to accelerated breaking of the Cu-O bond and enhanced selectivity for CH_3_OH.

### 4.2. Ethanol

Due to its high industrial value, ethanol has been receiving increasing attention in electrocatalytic ECR. The production of ethylene and ethanol often appears to be competitive, so it is important to effectively distinguish the reaction pathway to optimize their generation. Recently, CuO clusters supported on nitrogen-doped carbon nanosheets (Cu/N_0.14_C) were synthesized for ECR [99]. Under the potential of −1.1 V vs. RHE in 0.1 M KHCO_3_, a C_2+_ FE of 73% was achieved, including 51% ethanol production with a current density of −14.4 mA cm^−2^. It was revealed by means of operando XAS that CuO can transform to a Cu_n_-CuN_3_ moiety under catalytic ethanol production potential. Further operando FTIR showed a vibration at 1450 cm^−1^ when the potential was lower than −0.7 V vs. RHE, attributed to the antisymmetric methyl group vibration of CH_3_*, a critical intermediate for C_2_ formation. When the applied potential was below −1.1 V vs. RHE, surface-bound C=O species at ~1780 cm^−1^ and electrogenerated CO bound to the copper surface at 1920 cm^−1^ were found, suggesting that the adsorption of CO_2_ was the rate-determining step after CH_3_* formation. Combined calculation showed that the charge-asymmetric Cu_2_-CuN_3_ sites were intensified by CH_3_* adsorption, which strengthened the asymmetry of ethanol production.

**Figure 6:**
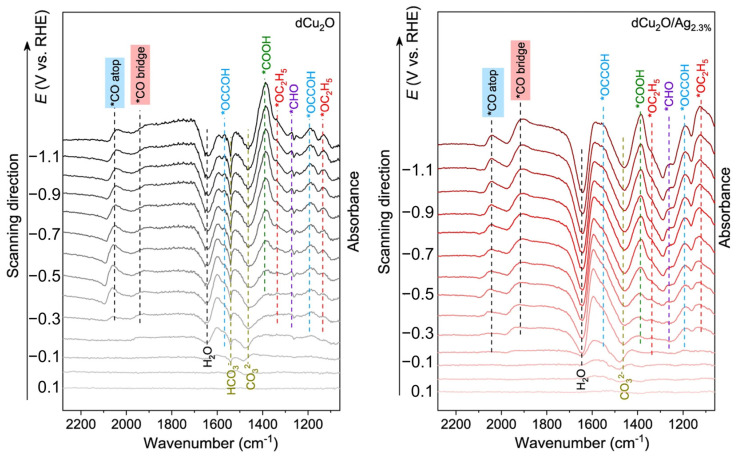
In situ ATR-IRAS obtained during chronopotentiometry in a potential window of 0.2 to −1.2 V vs. RHE for dCu_2_O (**left**) and dCu_2_O/Ag_2.3%_ (**right**) under ECR. Reproduced with permission from Ref. [101], Copyright (2022) Springer Nature.

The coexistence of different *CO adsorption configurations has been shown to be important for ethanol production, while doping strategies often make sense for regulating specific intermediates’ adsorption. For example, a K-doped Cu_2_Se nanosheet array on Cu foam was fabricated for ECR, achieving ethanol selectivity of over 70% at −0.8 V vs. RHE with 130 h stability [100]. In situ DRIFTS spectra were employed to explore the catalytic mechanism. Compared to pure Cu_2_Se, 11.2% K-doped Cu_2_Se exhibited different behavior. Specifically, the peak for the *CO_L_ intermediate gradually moved from 2084 cm^−1^ at 8 min to 2110 cm^−1^ at 14 min and then remained mostly stable. On the other hand, the peak for *CO_B_ shifted from 1698 cm^−1^ at 2 min to a higher wavenumber of 1708 cm^−1^ at 4 min, after which it remained constant. Contrarily, a redshift of *OH from 2 min to 6 min was also found. These results suggested the strengthened adsorption of *CO_L_ and *CO_B_ and the attenuated adsorption of *OH on the catalyst surface, which can promote ECR and suppress HER, respectively. After 2 h of electrolysis, only two intermediates of *CO_L_ and *OH were detected on Cu_2_Se, while all the intermediates were maintained on K11.2%-Cu_2_Se, elucidating the important role of K doping in keeping the carbonaceous intermediates on the catalyst surface, contributing to C-C coupling for ethanol production. The coexistence of *CO adsorption in atop and bridge configurations was also found via in situ ATR-IRAS in a silver-modified copper oxide system (dCu_2_O/Ag2.3%) to trigger asymmetric C-C coupling, achieving 40.8% selectivity for EtOH production. The Ag was thought to adjust the coordination number and oxidation state of surface Cu sites, steering the critical configuration of *CO adsorption [101] (Figure 6).

Besides doping, an optimized *CO adsorption strength and configuration can also be realized by means of surface modification to achieve selective C_2+_ production. Recently, Cu dendrites with a stable Cu^δ+^ state and hydrophobicity were synthesized via the surface coordination of carboxylate. The catalyst exhibited a C_2_ FE of 90.6% at a partial current density of 453.3 mA cm^−2^ in a flow cell and continuous production of C_2_H_5_OH solution with 90% relative purity at 600 mA over 50 h in a solid-electrolyte reactor. In situ Raman showed the bounded signal of *CO in both the atop and bridge sites on the catalyst, compared to the only CO_atop_ signal on Cu. The mixed CO adsorption configurations made the *CO dimerization process easier, promoting the conversion of CO_2_ to C_2_ products [102].

### 4.3. Propanol

Compared to that of ethanol and methanol, the efficient production of propanol via ECR seems to be more challenging due to the difficulty of the stabilization of C_1_ and C_2_ intermediates and C_1_-C_2_ coupling. But efforts have been made to explore effective strategies and gain a deeper understanding of the process of ECR to propanol.

A lithium electrochemical tuning approach was carried out to form high-density double sulfur vacancies in hexagonal CuS(100) planes, which was thought to enable the stabilization of CO* and OCCO* dimer and further coupling of CO-OCCO to form the key *C_3_ intermediate of n-propanol [103]. The FE of n-propanol production reached 15.4% in an H-cell. Recently, under supersaturated conditions in highly carbonated electrolytes, a CuAg alloy catalyst was proved to possess a high selectivity of 56.7% for 2-propanol production via ECR, with a specific current density of 59.3  mA  cm^−2^ [61]. In situ Raman was carried out to explore the *CO adsorption configuration under catalytic potential, with the finding that the ratio of *CO_bridge_ to *CO_atop_ linearly increased with [CO_2_] as the electrolyte enterd the supersaturated regime. A possible optimization of the proportion of *CO_bridge_ to *CO_atop_ to activate C-C coupling at a high *CO density on the catalyst surface was suggested by a volcano-shaped relationship centered around the optimal potential of −0.73 V vs. RHE between the *CO_bridge_-to-*CO_atop_ ratio, the FE of 2-propanol, and the applied potential. Moreover, the critical role of *OCH_2_CH_3_ was confirmed by isotopic labelling experiments. By adding hexadeuteroethanol (ethanol-d6) to the electrolyte, the 2-propanol-d8 formation rate was remarkably increased after electrocatalysis detected by NMR, suggesting *OCH_2_CH_3_ as the critical intermediate for C_1_-C_2_ coupling. Further, operando FTIR was performed and showed that as the potential decreased from −0.2 to −0.73 V vs. RHE, both the *CO and *OCH_2_CH_3_ bands were progressively intensified, suggesting higher formation rates of the intermediates. This was combined with a decrease in FE_CO_ and FE_ethanol_ and an increase in FE_2-propanol_. These results confirmed that under CO_2_-supersaturation conditions, the formation of 2-propanol instead of CO or ethanol was triggered by the high densities of *CO and *OCH_2_CH_3_ intermediates. Calculations suggested that the surface binding of intermediates in the middle position of the alkyl chain and the C-O bonds were weakened and strengthened, respectively, due to the dispersed Ag atoms in Cu, favoring the formation of 2-propanol over 1-propanol.

## 5. Summary and Perspectives

In conclusion, this review focused on the conversion of CO_2_ to alcohols using Cu-based catalysts, and several strategies have been proposed to achieve efficient production of alcohols. The highest selectivity values obtained for catalysts for ECR to alcohols in recent years are summarized in Table 2. We provided a systematic discussion of the mechanisms involved in CO_2_-to-alcohol conversion and highlighted the in situ/operando advanced spectroelectrochemical analysis techniques for alcohol selectivity. We delved into the structural features of Cu-based catalysts, ranging from the surface to the interface, to gain a deeper understanding of the factors influencing alcohol formation. By examining and analyzing these factors, we aimed to uncover valuable insights that can contribute to the development of more efficient Cu-based catalysts for selective alcohol production from CO_2_. This knowledge will play a crucial role in advancing the field of CO_2_ reduction and promoting sustainable and carbon-neutral fuel production. However, there are several areas that require further attention and development to enable the practical application of these catalysts for efficient ECR:

1. It is important to conduct more research on constructing model systems to study the structure–activity relationship of catalysts in ECR alcohol production more rigorously and clearly. Additionally, the development of more advanced in situ/operando techniques with higher spatiotemporal resolution is necessary to obtain more localized information about the catalytic system (e.g., AFM-IR and tip-enhanced Raman spectroscopy [104,105,106,107]). Traditional spectroelectrochemical techniques often lack spatial resolution, which limits our detailed understanding of different catalyst components.
2. It is crucial to pay more attention to propanol and alcohols with longer carbon chains due to their high value and relatively limited understanding. The stability of the catalysts should also be taken into consideration for their practical application, in addition to their catalytic activity.
3. Efforts should be made to design reactors with higher efficiency for ECR. For example, incorporating membrane electrode assembly (MEA) can enhance the performance of catalysts and improve overall efficiency [108,109].
4. The integration of artificial intelligence (AI) and density functional theory (DFT) simulations can be utilized to predict and identify the best catalysts for alcohol production through CO_2_ reduction [110,111]. This approach will aid in the development of more efficient electrocatalytic ECR processes.
5. The literature has primarily focused on electrocatalyst design, but it has become evident that the same electrocatalysts can yield different products and selectivity depending on whether they are in contact with the bulk electrolyte. For example, “gas-phase” operations (also known as electrolyte-less conditions or zero gap) favor the formation of ethanol compared to “liquid-phase” operations with copper-based (Cu_x_O) gas diffusion electrodes [112,113,114]. The exact reason for this difference in terms of the working state during electrocatalytic operations is still unclear. In future research, mechanistic studies on C_2+_ formation, especially alcohols, should account for the effects of the electrolyte, CO_2_ diffusion to the electrocatalyst, the concentration of adspecies on the electrode surface, and how these aspects are influenced by the application of an electrical potential.

By addressing these aspects, the field of electrocatalytic CO_2_ reduction can be advanced and pave the way for practical applications in sustainable alcohol production and carbon neutralization.

## Figures and Tables

**Table 1:** Electrode Reactions with Equilibrium Potentials (V vs. RHE) [13].

Product	Reaction	Potential
Methanol	CO_2_(g) + 6H^+^ + 6e^−^ → CH_3_OH (l) + H_2_O (l)	0.03
Ethanol	2CO_2_(g) + 12H^+^ + 12e^−^ → C_2_H_5_OH (l) + 3H_2_O (l)	0.09
Propanol	3CO_2_(g) + 18H^+^ + 18e^−^ → CH_3_CH_2_CH_2_OH (l) + 5H_2_O (l)	0.1

**Table 2:** Summary of the Cu-based catalysts for ECR to alcohols.

Product	Catalysts	Electrolyte	Cell	Active Sites	Performance	Ref.
Methanol	Ag, S-Cu_2_O/Cu	1-butyl-3-methylimidazolium tetrafluoroborate/H_2_O	H cell	Dual-doped porous Cu_2_O/Cu	−1.18 V vs. RHE, FE 67.4%, −122.7 mA cm^−2^	[97]
	Pd_83_Cu_17_	25 mol% [Bmim]BF_4_ + 75 mol% water	H cell	Pd/Cu grain boundaries with high Pd^0^ /Pd^II^ and Cu^I^ + Cu^0^ /Cu^II^ ratios	−2.1 V vs. Ag/Ag^+^, FE 80%, 31.8 mA cm^−2^	[60]
	CuSAs/TCNFs	0.1 M KHCO_3_	H cell	Cu single atoms with high binding energy for *CO intermediate	−0.9 V vs. RHE, FE 44%, −92 mA cm^−2^	[72]
Ethanol	TWN-Cu_x_-600- SACs	0.5 M CsHCO_3_	H cell	Adjacent Cu−N_3_ sites	−1.1 V vs. RHE, FE 81.9%, 35.6 mA cm^–2^	[80]
	Alkanethiol-modified sputtered copper	1 M KOH	Flow cell	Cu with tailored interfacial wettability	−1.2 V vs. RHE, FE 53.7%	[84]
	Cu-Li	0.1 M KHCO_3_	RDE cell	In situ formed Cu_n_ clusters	−0.7 V vs. RHE, FE 91%	[74]
	Cu/N_0.14_C	0.1 M KHCO_3_	H cell	Charge-asymmetry Cu_2_-CuN_3_ clusters	−1.1 V vs. RHE, FE 51%, −14.4 mA cm^−2^	[99]
	K-doped Cu_2_Se	0.1 M KHCO_3_	H cell	Stabilized Cu^I^ species	−0.8 V vs. RHE, FE 70.3%, −35.8 mA cm^−2^	[100]
Propanol	CuAg alloy	1 M CsHCO_3_	High-pressure reactor	Cu with dispersed Ag atoms	−0.7 V vs. RHE, FE 59.3%, 56.7 mA cm^−2^	[61]
	CuS_x_	0.1 M KHCO_3_	H cell	CuS_x_ with double sulfur vacancies	−1.05 V vs. RHE, FE 15.4%, 3.1 mA cm^−2^	[103]

## Data Availability

Not applicable.
